# Feasibility of a Smartphone-Based Hearing Aid App for Mild-to-Moderate Hearing Loss: Prospective Multicenter Randomized Controlled Trial

**DOI:** 10.2196/46911

**Published:** 2023-10-05

**Authors:** Sungmin Jo, Moo Kyun Park, Jae-Hyun Seo, Ki-eun Lee, Jae Sang Han, Ji Hyung Lim, Jun Ho Lee, Seung-Ha Oh

**Affiliations:** 1 Department of Otorhinolaryngology-Head and Neck Surgery, Seoul National University Hospital, Seoul, Republic of Korea; 2 Department of Speech Pathology and Audiology, Graduate School, Hallym University, Chuncheon, Republic of Korea; 3 Sensory Organ Research Institute, Seoul National University Medical Research Center, Seoul, Republic of Korea; 4 Department of Otorhinolaryngology-Head and Neck Surgery, College of Medicine, The Catholic University of Korea, Seoul, Republic of Korea

**Keywords:** hearing aid apps, hearing aids, hearing loss, prospective, smartphone, hearing rehabilitation

## Abstract

**Background:**

Hearing loss is a growing health concern worldwide. Hearing aids (HAs) are the treatment of choice for hearing rehabilitation in most cases of mild-to-moderate hearing loss. However, many patients with hearing loss do not use HAs due to their high cost, stigma, and inaccessibility. Since smartphones are widely used, many apps that mimic the amplification function of HAs have been introduced. Smartphone-based HA apps (SHAAs) are affordable and easy to access. However, the audiological benefit of SHAAs has not been determined.

**Objective:**

We compared the audiological performance between an SHAA and a conventional HA in a prospective, multicenter randomized controlled trial.

**Methods:**

Patients with mild-to-moderate hearing loss were prospectively enrolled from 2 tertiary hospitals and randomly assigned to either an SHAA (Petralex; IT4YOU Corp LLC) or a conventional HA (Siya 1 miniRITE; Oticon A/S). For the cross-over study design, participants used the alternate device and repeated the same 2-month trial. Audiological measurements were obtained using hearing tests, real-ear measurements, and the hearing-in-noise test (HINT). Subjective satisfaction was evaluated using the Abbreviated Profile of Hearing Aid Benefit (APHAB) and International Outcome Inventory for Hearing Aids (IOI-HA).

**Results:**

Overall, 63 participants were screened and 38 completed the study. In sound-field audiometry testing, the SHAA showed a 20- to 60-dB gain in the low-to-high frequencies of the hearing threshold level. The HA provided adequate gain in the middle-to-high frequencies (55, 65, and 75 dB in real-ear measurements), which is the sound level for most speaking volumes. However, the SHAA could not improve word recognition at 50 dB. The HA showed better audiological performance than the SHAA in both quiet and noisy conditions in the HINT. The IOI-HA scores were significantly improved by both the HA and SHAA versus unaided conditions. Among the SHAA users, 37% (14/38), 42% (16/38), 24% (9/38), and 32% (12/38) showed improvement in APHAB scores for ease of communication, reverberation, background noise, and aversiveness of sounds, respectively. There were no differences in adverse events between the 2 study groups.

**Conclusions:**

The HA showed better performance than the SHAA in word recognition and the HINT. However, the SHAA was significantly better than unaided hearing in terms of amplification. The SHAA may be a useful hearing assistance device for patients with mild-to-moderate hearing loss when listening to soft sounds in quiet conditions. The SHAA demonstrated poorer performance than the HA in the mid- to high-frequency sounds that are important for word recognition, sound quality, and hearing in noisy conditions. Further development of the signal technology of SHAAs is needed to improve the sound quality of mid- to high-frequency sounds and overcome noisy environments.

## Introduction

Hearing loss is a common and growing global health issue. The World Health Organization (WHO) reported that approximately 2.5 billion people will experience hearing loss and 700 million will need hearing rehabilitation by 2050 [1]. The prevalence of hearing loss increases with age [2]. Further, 9 in 10 people with hearing loss are adults and >70% are older adults. Among those aged >60 years, one-fourth have hearing loss [3]. Patients with hearing loss have difficulty communicating and understanding speech [4]. Patients with hearing loss often perform poorly in school and have a higher unemployment rate [5,6]. Hearing loss decreases quality of life and cognitive function, as well as increasing anxiety and depression. Hearing loss is the largest population-attributable fraction for dementia [7]. The WHO suggests that the annual global cost for unaddressed hearing loss is approximately US $980 billion [8].

Hearing rehabilitation improves audiological performance, daily activity functioning, and quality of life. Hearing aids (HAs) are the most common and important method of hearing rehabilitation [9,10]. Conventional HAs have shown significantly improved amplification in patients with mild-to-moderate hearing loss [11]. HAs benefit physical, social, emotional, and mental well-being [12]. However, due to the global high costs, stigma, difficult accessibility, and inconvenience, only 17% of those who need HAs for hearing loss have them [13]. People who have internalized a strong sense of stigma against hearing loss can exhibit denial of the condition, leading them to delay or reject hearing habilitation interventions [14]. Other barriers include low levels of social support from family and friends, which is an important factor in empowering patients to receive hearing rehabilitation [15]. Furthermore, the HA utilization rate is primarily affected by HA prices and insurance reimbursement policies [16]. In South Korea, the proportion of people receiving HA subsidies increased after reimbursement for HAs increased [16]. HAs are used by only 6% of the population in India and 9% in Peru [17], whereas in other low-income countries, HA use is almost nonexistent. Recently, the US government approved over-the-counter HAs to improve access and provide HAs at a lower cost [18]. Hearing loss is the dementia cause with the largest population-attributable fraction, and HA use is the most important factor protecting against cognitive decline [1].

Smartphones can be useful devices for amplification, and many smartphone-based HA apps (SHAAs) have been developed to improve the audibility of sounds for both individuals with and without hearing impairment [19]. They use signal-processing algorithms similar to those used in conventional HAs. Accessibility and low cost are the main advantages of SHAAs [20]. They are either free or require a subscription after a free trial. The subscription fee is very low compared to HA costs and even to other personal sound amplification devices [21,22]. For example, the subscription fee of the Petralex HA app is currently US $12.43/mo or US $59.50/y, but the retail price of the Siya 1 miniRITE HA is US $2863/piece. Smartphone users can easily locate and download these apps from their internet store. SHAAs allow users to access hearing amplification through earbuds or headphones. The basic features of SHAAs include adjustable amplification, an equalizer, and sometimes a noise reduction feature. Other options include self-audiometry via earbuds and feedback control. However, if a patient wants to purchase an HA, receive follow-up to control sound quality, and benefit from various advanced functions such as noise reduction, visiting a specialist is necessary. A growing number of individuals with hearing impairment who are not ready to invest in conventional HAs have shown an interest in SHAAs due to their accessibility, ease of use, and low cost [19]. SHAA use may also improve a person’s attitude toward amplification and conventional HAs [23].

Although some SHAAs provide the recommended level of processing delay, conventional HAs have shown better performance than SHAAs in most aspects of amplification. Our previous study on the electroacoustic performance of various SHAAs [19] demonstrated that some SHAAs provided satisfactory amplification [24]. In addition, other studies have demonstrated the feasibility of SHAAs for patients with hearing loss [24]. In this study, we investigated the listening performance of an SHAA using objective assessments and subjective satisfaction in patients with mild-to-moderate hearing loss.

This study compared the audiological performance of an SHAA to that of a conventional HA. The study settings included unaided and aided conditions in quiet and noisy situations. The objective benefit of hearing amplification was evaluated in sound-field tests and real-ear measurements (REMs). We used the sound-field test to evaluate the hearing threshold and word recognition scores (WRSs) at 50 dB. REMs were studied using 55-, 65-, and 75-dB stimuli in the 250-8000 Hz range. The subjective benefits were evaluated using the Abbreviated Profile of Hearing Aid Benefit (APHAB) and International Outcome Inventory for Hearing Aids (IOI-HA).

## Methods

### Study Design

A prospective randomized controlled trial was conducted at 2 tertiary hospitals in South Korea (the Department of Otolaryngology, Seoul National University Hospital and the Department of Otolaryngology, The Catholic University of Korea, Seoul St. Mary’s Hospital) from August 2020 to November 2022. The inclusion criteria were (1) acquired symmetrical, sensorineural, and mild-to-moderate hearing loss; (2) current use of a smartphone; and (3) no previous experience with hearing amplification. Mild and moderate hearing loss ranged from the 26- to 40-db hearing level and from the 41- to 55-dB hearing level, respectively (according to the American Speech-Language-Hearing Association) [25], on pure-tone averages of 0.5, 1, 2, and 4 kHz. Patients with communication problems, central nervous system diseases, lesions of the auditory nerve, or external and middle ear diseases or anomalies were excluded.

To identify sensorineural hearing loss, otoscopy, pure-tone audiometry (PTA) by air and bone conduction, and tympanometry were performed. All participants visited 4 times and all tests were conducted in a sound-treated room. At the first visit, demographic assessment, otoscopy, tympanogram, tinnitogram, PTA, and speech audiometry were conducted. At the second visit, the participants who passed the screening test were enrolled; otoscopy, sound-field unaided and aided PTA, and WRS testing were performed; and our questionnaire was completed. At this time, the participants were randomly given their first hearing amplification to use for 2 months. At the 2-month visit, the same tests were conducted except for unaided PTA and WRS testing. The hearing amplification method was then switched to be used for another 2 months. At the final visit, participants completed the same tests as in the previous visit. Hearing impairment (>20-dB change in 1 frequency, >10-dB change in 2 consecutive frequencies, or >5-dB change in 3 consecutive frequencies) and tinnitus (>1 mo for at least 8 h/d) were considered adverse events in this study.

### Ethical Considerations

This study was registered in the clinical trials registry in South Korea (Clinical Research Information Service; KCT0005458) [26] and at ClinicalTrials.gov (NCT05644106). This study was approved by the Institutional Review Board of Seoul National University Hospital (No. 2003-028-1109), Seoul, South Korea. Informed consent was obtained from all participants.

### Hearing Amplification Systems (HA and SHAA)

A pilot study that conducted behavioral evaluations of 3 SHAAs provided the evidence used to select an SHAA for this study [24]. We chose the Petralex SHAA (IT4YOU Corp LLC) because it was available for download on both Android and iOS phones and users showed a greater improvement in WRSs compared to the other apps [24]. Petralex provides various functions, including a hearing test, speech recognition, acoustic amplification, and dynamic compression. It can amplify sound up to 30 dB, and the gain can be adjusted based on the hearing test results. In addition, a noise reduction function reduces background noise and focuses on speech recognition.

For the conventional HA, we used the Siya 1 miniRITE (Oticon A/S), coupled with two 85-dB receivers and single closed ear tips. The Siya 1 miniRITE is an essential-level HA, first introduced in 2018 with 48 channels and 10 adjustable bands. The Siya 1 miniRITE has basic modern digital HA technology including advanced feedback management, noise reduction, and multiband directionality. During the study, special functions of the Siya 1 miniRITE were activated, including instantaneous noise management, binaural bandwidth processing, and a feedback shield. However, noise reduction was not activated. All participants used each method, alternating at 2 months. Before the study, all measurement materials were calibrated.

### Sound-Field Audiometry and WRS Tests

Sound-field audiometry (SFA) and WRS tests were performed using a calibrated Interacoustics AC40 (Interacoustics A/S). To determine the difference between the SHAA and the conventional HA, SFA was obtained at 0.25, 0.5, 1, 2, 3, 4, 6, and 8 kHz for both hearing amplification systems. Warble tone was used to avoid standing waves. The WRS was evaluated using the Korean Standard Monosyllabic Word List for Adults [27], which comprised 25 monosyllabic words per condition and was presented at the most comfortable sound level. Lastly, the mean and SD of the WRS were calculated under unaided and aided conditions to measure the improvement. The WRS test in an aided condition was presented at a hearing level of 50 dB.

### REM Test

This test measures actual HA gain by comparing the difference between the HA target value and the sound pressure measured using a microphone probe tip in the ear canal. We used Affinity 2.0 software (version 2.6.0; Serif Ltd) to conduct the REMs. To validate the HA performance, the REMs were performed using speech stimuli divided into a soft level (55-dB sound pressure level [SPL]), a medium level (65-dB SPL), and a loud level (75-dB SPL) based on the National Acoustic Laboratory–NL2 prescription. For the SHAA, the gain control and parameters were modified manually to match the targets.

### Hearing-in-Noise Test

The hearing-in-noise test (HINT) was developed to measure binaural speech recognition ability under quiet and noisy situations [28]. We used the Korean version of the HINT, which is composed of 12 lists with 20 sentences per list [29]. Tests were performed under 4 conditions: quiet, front noise, left noise, and right noise. The participants were positioned 1 m in front of the speaker, and the speech or noise was first presented at a 65-dB SPL (0-dB signal-to-noise ratio [SNR]) through the speaker. If the participant responded correctly, we adjusted the level of the subsequent sentence using the transformed up-down methods recommended by Levitt [30] in 1971. When the listener achieved 50% of correct answers, the SNR was fixed at the corresponding level. These measurements were conducted using both hearing amplification methods.

### Questionnaires

For the measurement of subjective satisfaction, the APHAB and IOI-HA were used. The APHAB consists of 4 items: ease of communication, reverberation, background noise, and aversiveness of sounds. Each item has 6 questions, and higher scores indicate greater disability. This survey can be used to evaluate the performance of an HA in unaided and aided conditions. The IOI-HA is a self-estimation tool designed to check the efficiency of HA fitting [31]. It contains 7 items including use of the HA, benefit of the HA, residual activity limitations, satisfaction, residual participation restrictions, impact on others, and quality of life.

### Statistical Analysis

A sample size of 26 would achieve 90% power to detect noninferiority with a 1-sided significance level of .025, and a cross-over design would have a margin of noninferiority of −10%, a true mean difference of 0, and an SD of the paired differences of 15, based on the test results from a previous study for understanding speech in noise [32]. Therefore, we aimed to enroll 33 participants with the anticipation of a 20% dropout rate. Although the noninferiority margin for otolaryngological interventions has not been established, a noninferiority margin of 10% is commonly used for drugs intended for treatment. Continuous variables were expressed as mean (SD), and all statistical analyses were performed using SPSS (version 25.0; IBM Corp). The 2-tailed *t* test was used to compare continuous variables, and one-way ANOVA with the Scheffé post hoc test was used to compare the questionnaire scores and HINT performances of 3 groups, including in unaided conditions. One-way ANOVA *P* values of <.05 were considered to indicate statistical significance.

## Results

### Participants

**Figure 1.**
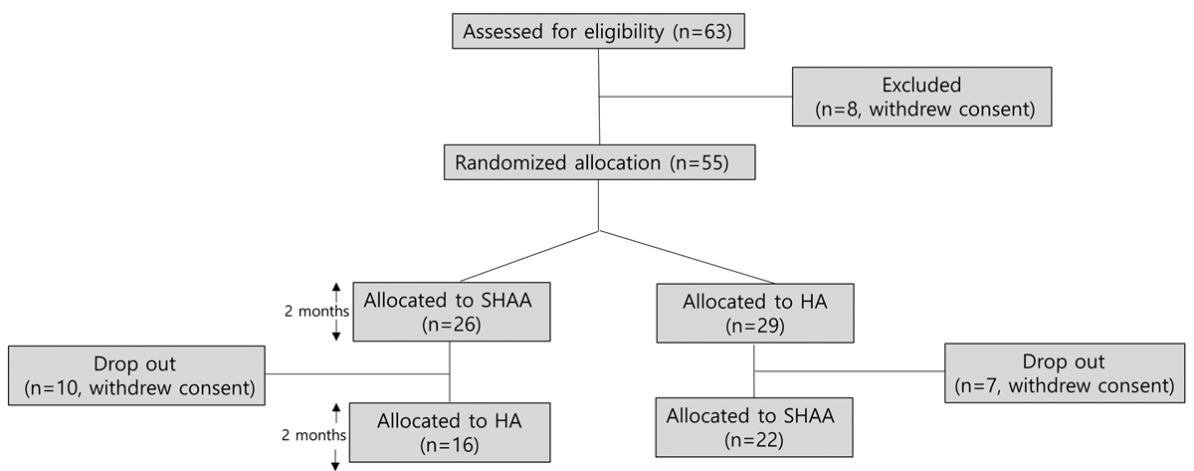
Flow diagram of the study design. HA: hearing aid; SHAA: smartphone-based hearing aid app.

A total of 63 participants were screened; 8 participants were excluded due to withdrawal of consent and greater than mild-to-moderate hearing loss. During the 2-month follow-up, 10 participants in group A dropped out for personal reasons and 7 participants in group B dropped out because they withdrew consent. The final analysis was performed on 38 participants (Figure 1). REMs and the HINT were performed in 25 (66%) out of 38 participants. The mean age of the study participants was 66 (range 41-84) years, and 42% (16/38) were male. Most participants were without tinnitus (22/38, 58%), and the degree of hearing loss was mild (23/38, 61%) to moderate (15/38, 39%). The characteristics of the participants are shown in Table 1. Although SHAAs may have potential adverse effects, such as tinnitus and discomfort, because patients can increase the SHAA volume to a risky sound level [33], there were no serious complications (hearing impairment or tinnitus) in either group during this study.

**Table 1.** Characteristics of the participants.

Characteristics		Values (n=38)
Age (years), mean (SD)		66 (12)
**Sex, n (%**)		
	Male	16 (42)
	Female	22 (58)
**Tinnitus, n (%**)		
	Yes	16 (42)
	No	22 (58)
**Hearing loss (dB HL**^a^)		
	4-frequency average^b^, mean (SD)	39 (7)
	26-40, n (%)	23 (61)
	41-55, n (%)	15 (39)
Word recognition score, mean (SD)		66 (20)

a dB HL: decibel of hearing level.

b 4 frequency averages: 500, 1000, 2000, and 4000 Hz.

### SFA and WRS Results

**Figure 2.**
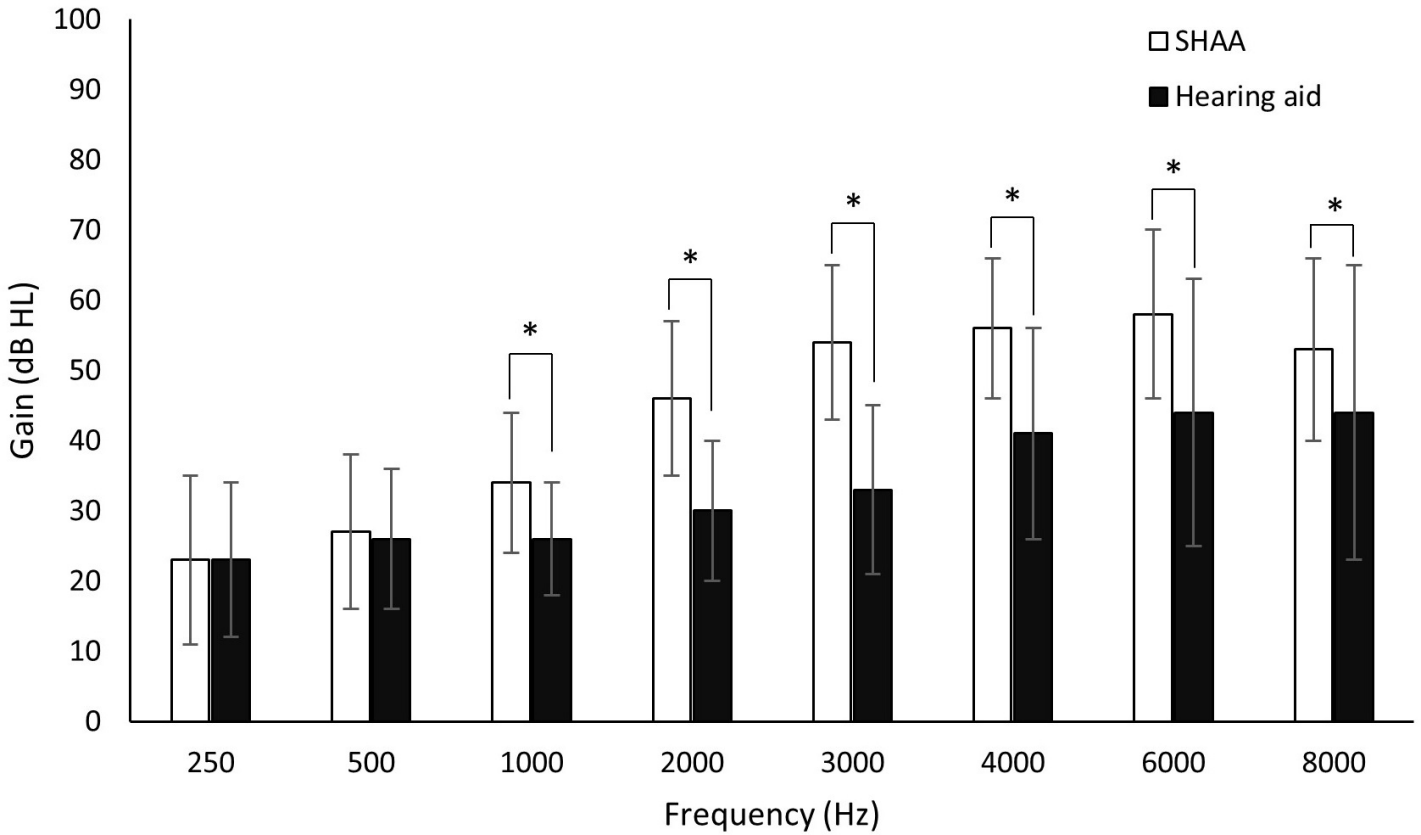
Sound-field (warble-tone) audiometry thresholds in the HA and SHAA groups. Error bars indicate the SD. db HL: decibel of hearing level; HA: hearing aid; SHAA: smartphone-based hearing aid app. *P<.05.

**Figure 3.**
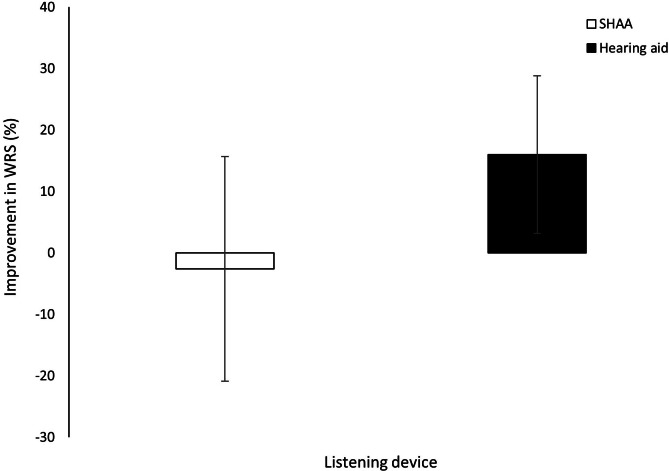
Improvements in WRS, comparing unaided and aided conditions at 50-dB hearing level stimuli. Error bars indicate the SD. SHAA: smartphone-based hearing aid app; WRS: word recognition score.

The SHAA showed a slightly lower gain at all frequencies than the conventional HA, with statistical significance at 1000 Hz (*P*=.001), 2000 Hz (*P*<.001), 3000 Hz (*P*<.001), 4000 Hz (*P*<.001), 6000 Hz (*P*=.001), and 8000 Hz (*P*=.04; Figure 2). The mean improvement in WRS was −2.6 (SD 18.3) and 16.0 (SD 12.8) for the SHAA and HA groups, respectively (*P*<.001). Most participants (24/38, 63%) showed no improvement with the SHAA compared to unaided conditions (Figure 3).

### REM Results

**Figure 4.**
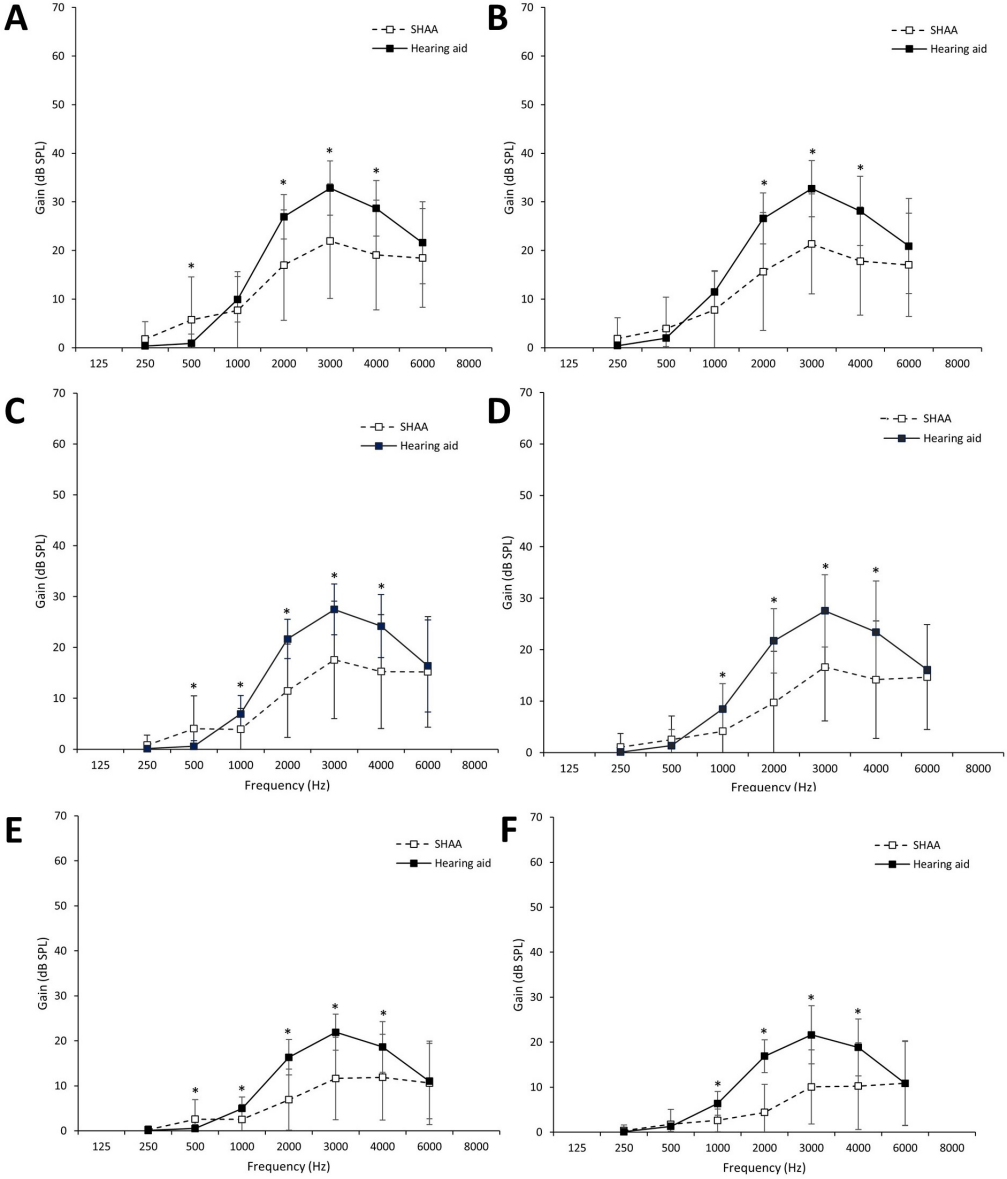
Real-ear aided responses (REARs) of the hearing aid and SHAA groups measured at the 55-, 65-, and 75-dB sound pressure levels (SPLs): average REAR in the right ear at the (A) 55-, (B) 65-, and (C) 75-dB SPLs and average REAR in the left ear at the (D) 55-, (E), 65-, and (F) 75-dB SPLs. Error bars indicate the SD. SHAA: smartphone-based hearing aid app. *P<.05.

In real-ear aided responses at 55 dB, the HA showed a larger gain at 1000, 2000, 3000, 4000, and 6000 Hz than the SHAA. The SHAA showed a larger gain at low frequencies (250 and 500 Hz) than the HA. Statistical significance was found at 500 Hz (*P*=.01), 2000 Hz (*P*<.001), 3000 Hz (*P*<.001), and 4000 Hz (*P*=.001) in the right ear and at 2000 Hz (*P*<.001), 3000 Hz (*P*<.001), and 4000 Hz (*P*<.001) in the left ear. At 65 dB, the HA revealed greater gains at 1000, 2000, 3000, 4000, and 6000 Hz than the SHAA. Statistical significance was found at 500 Hz (*P*=.02), 1000 Hz (*P*=.01), 2000 Hz (*P*<.001), 3000 Hz (*P*=.001), and 4000 Hz (*P*=.002) in the right ear and at 1000 Hz (*P*<.001), 2000 Hz (*P*<.001), 3000 Hz (*P*<.001), and 4000 Hz (*P*=.002) in the left ear. At 75 dB, the HAs showed a larger gain at 1000, 2000, 3000, and 4000 Hz than the SHAA. As with the results at 65 db, statistical significance was found at 500 Hz (*P*=.03), 1000 Hz (*P*=.007), 2000 Hz (*P*<.001), 3000 Hz (*P*<.001), and 4000 Hz (*P*=.004) in the right ear and at 1000 Hz (*P*<.001), 2000 Hz (*P*<.001), 3000 Hz (*P*<.001), and 4000 Hz (*P*=.001) in the left ear. At all speech levels, the SHAA showed greater gain at low frequencies (250 and 500 Hz) than the HA (Figure 4).

### HINT Results

**Figure 5.**
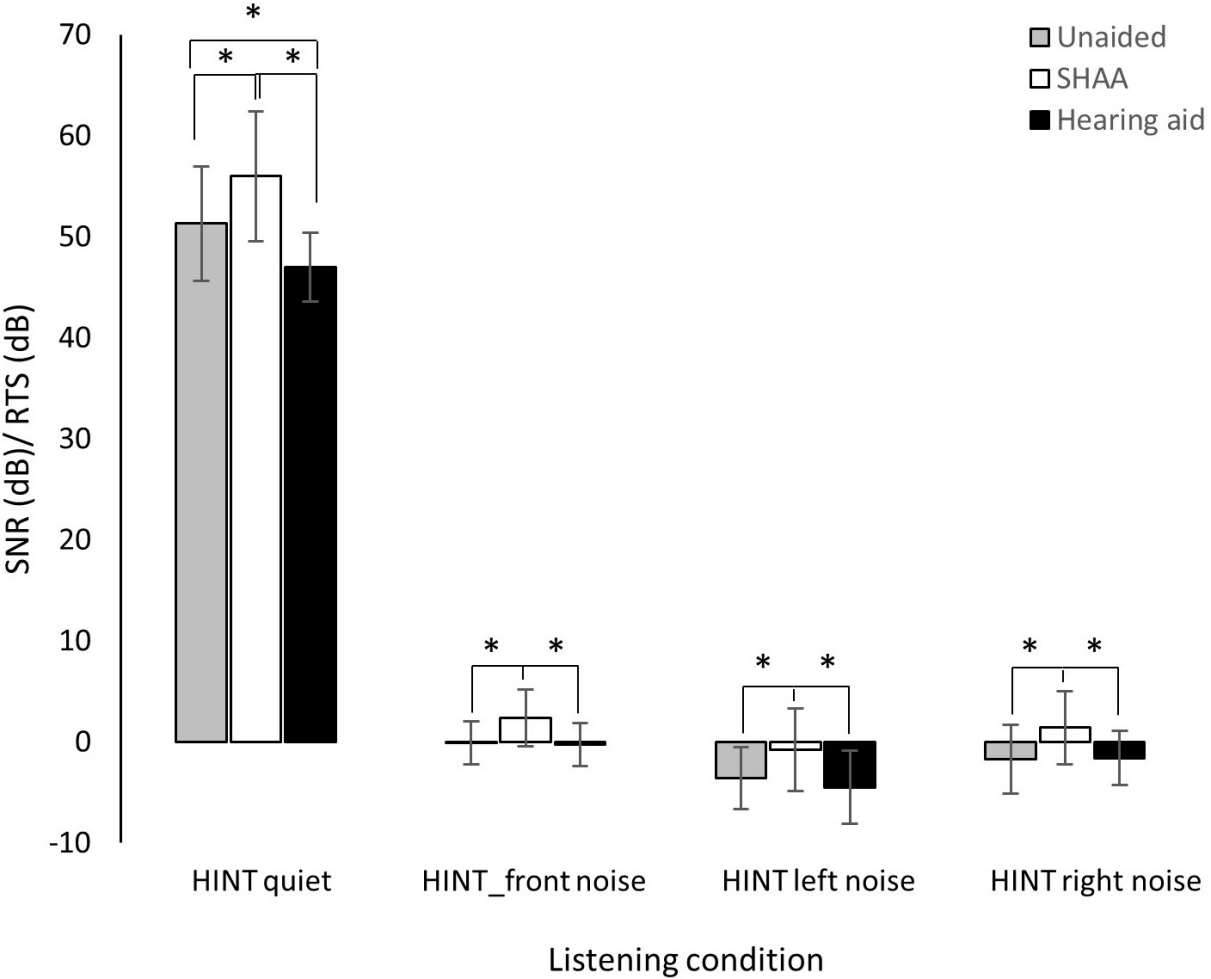
Hearing-in-noise test (HINT) in quiet, front noise, left noise, and right noise conditions for the unaided, hearing aid, and SHAA groups. Error bars indicate the SD. RTS: reception threshold for speech; SHAA: smartphone-based hearing aid app; SNR: signal-to-noise ratio.

The mean SNRs (HINT performance) in the unaided, HA, and SHAA groups were 51.3 (SD 5.6), 47 (SD 3.4), and 56 (SD 6.4) dB, respectively, in a quiet situation (*F*_2,72_=17.164; *P<*.001). The Scheffé post hoc analysis showed a statistically significant difference between the unaided and SHAA groups (*P*=.006), the unaided and HA groups (*P*=.046), and the SHAA and HA groups (*P*<.001). The mean SNR (performance) was −0.1 (SD 2.1), –0.3 (SD 2.1), and 2.4 (SD 2.8) dB in a front noise situation and −1.7 (SD 3.4), –1.6 (SD 2.7), and 1.4 (SD 3.6) dB in a right noise situation (*F*_2,72_=7.110; *P*=.002) for the unaided, HA, and SHAA groups, respectively. In the Scheffé post hoc analysis, there were statistically significant differences between the HA and SHAA groups (*P*=.009) and between the unaided and SHAA groups (*P*=.006; Figure 5). The mean SNR (performance) was −3.6 (SD 3.0), –4.5 (SD 3.6), and −0.8 (SD 4.1) dB in a left noise situation for the unaided, HA, and SHAA groups, respectively, showing statistical significance (*F*_2,72_=7.008; *P*=.002)

### Questionnaire Results

**Figure 6.**
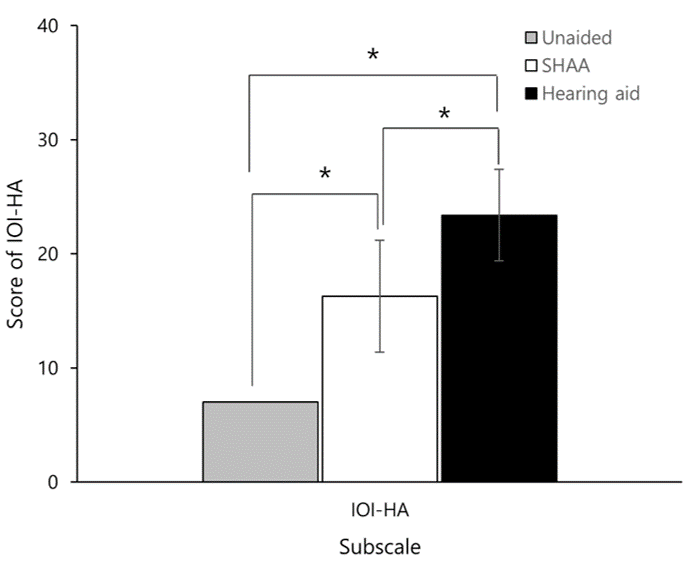
International Outcome Inventory for Hearing Aids (IOI-HA) items for the unaided, hearing aid, and SHAA groups. Error bars represent the SD. SHAA: smartphone-based hearing aid app. *P<.05.

The mean score of the IOI-HA was 7 (SD 0), 23.4 (SD 4.0), and 16.3 (SD 4.9) in the unaided, HA, and SHAA groups, respectively (*F*_2,111_=187.469; *P*<.001). The Scheffé post hoc analysis showed statistically significant differences between all groups (*P*<.001). The HA group scored the highest, followed by the SHAA and unaided groups (Figure 6).

**Figure 7.**
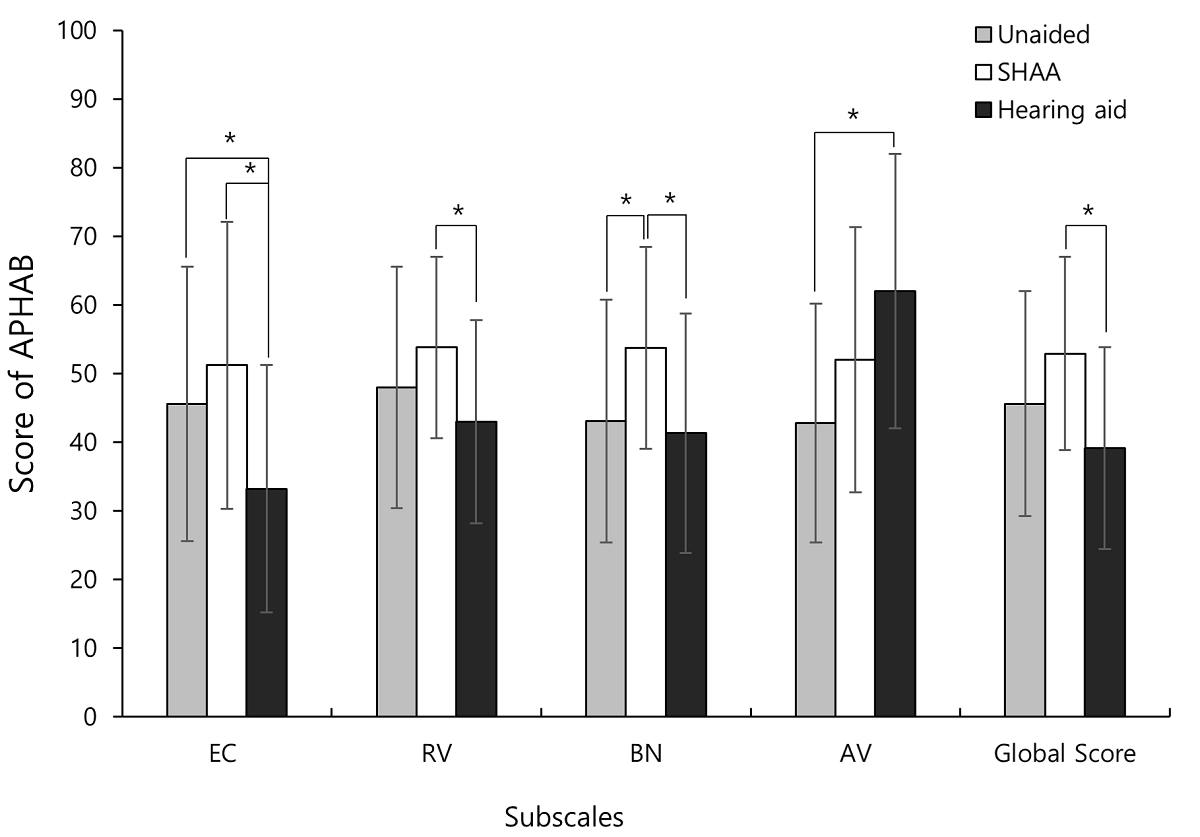
Mean score of the Abbreviated Profile of Hearing Aid Benefit (APHAB) items for the unaided, hearing aid, and smartphone-based hearing aid app (SHAA) groups. Error bars indicate the SD. AV: aversiveness of sounds; BN: background noise; EC: ease of communication; RV: reverberation. *P<.05.

In the ease of communication subscale item of the APHAB, the mean scores were 45.6 (SD 20.0), 51.2 (SD 20.9), and 33.2 (SD 18.0) in the unaided, SHAA, and HA groups, respectively (*F*_2,111_=8.009; *P*=.001). The Scheffé post hoc analysis showed statistically significant differences between the unaided and HA groups (*P*=.03) and between the HA and SHAA groups (*P*=.001). The HA group had the lowest scores, followed by the unaided and SHAA groups. In the reverberation subscale item of the APHAB, the mean scores were 48.0 (SD 17.6), 53.8 (SD 13.2), and 43.0 (SD 14.8) in the unaided, SHAA, and HA groups, respectively (*F*_2,111_=5.525; *P*=.006). The Scheffé post hoc analysis indicated that the mean score of the HA group was significantly lower than that of the SHAA group (*P*=.01). In the background noise subscale item of the APHAB, the mean scores were 43.1 (SD 17.7), 53.7 (SD 14.7), and 41.3 (SD 17.4) in the unaided, SHAA, and HA groups, respectively (*F*_2,111_=6.010; *P*=.003). After the Scheffé post hoc analysis, significant differences were observed between the unaided and SHAA groups (*P*=.03) and between the HA and SHAA groups (*P*=.007). The SHAA group had the highest scores, followed by the unaided and HA groups. In the aversiveness of sound subscale of the APHAB, the mean scores were 42.8 (SD 17.4), 52.0 (SD 19.3), and 62.0 (SD 20.0) in the unaided, SHAA, and HA groups, respectively (*F*_2,111_=9.509; *P*<.001). The Scheffé post hoc analysis indicated that the mean score of the unaided group was significantly lower than that of the HA group (*P*<.001). In particular, the SHAA group’s score was lower than that of the HA group, but without statistical significance (*P*=.08). Lastly, the mean global scores were 45.6 (SD 16.4), 52.9 (SD 14.1), and 39.1 (SD 14.7) in the unaided, SHAA, and HA groups, respectively (*F*_2,111_=7.689; *P*=.001). The Scheffé post hoc analysis showed a significant difference between the HA and SHAA groups (*P*<.001; Figure 7).

A comparison of individuals in the SHAA group between unaided and aided conditions found that 37% (14/38), 42% (16/38), 24% (9/38), and 32% (12/38) of users showed improvements in the ease of communication, reverberation, background noise, and aversiveness of sound APHAB subscales, respectively. In the same subscales of the APHAB, the mean postintervention scores were 43.1 (SD 24.0), 50.2 (SD 10.8), 45.8 (SD 8.0), and 35.4 (SD 16.1), respectively (Table 2).

**Table 2.** Mean scores of the Abbreviated Profile of Hearing Aid Benefit (APHAB) items for the unaided and aided conditions in the SHAA group, with improvements in ease of communication, reverberation, background noise, and aversiveness of sound.

Subscale	Participants (n=38), n (%)	Pretest score, mean (SD)	Posttest score, mean (SD)
Ease of communication	14 (37)	57.1 (20.4)	43.1 (24.0)
Reverberation	16 (42)	60.6 (9.6)	45.8 (8.0)
Background noise	9 (24)	57.6 (8.2)	50.2 (10.8)
Aversiveness of sound	12 (32)	53.3 (9.4)	35.4 (16.1)

## Discussion

### Principal Findings

This study used a noninferiority study design to compare the audiological performance between an SHAA and a conventional HA. The SHAA did not show noninferiority to the HA in the WRS test, which was the primary measurement used in the original study. However, the SHAA showed feasibility in some users, especially in unaided conditions and for the amplification of soft sounds. In this study, 63 participants were screened and 38 finished the study. The objective audiological performance was evaluated using the warble-tone hearing test, the WRS test, REMs, and the HINT, and subjective satisfaction was evaluated using the APHAB and IOI-HA questionnaires. The aided hearing thresholds for the SHAA were 20-60 dB, from low to high frequencies. Although the SHAA group showed a lower gain at the 1000- to 8000-Hz levels than the HA group, it showed gains similar to those of the HA group at low frequencies. The REMs results at 75 dB, as well as the common speaking levels of 55 and 65 dB, indicated that the HA group showed greater gains in the middle-to-high frequencies than the SHAA group. One-third (14/38, 37%) of the SHAA group showed slight improvements in WRS. However, most SHAA users (24/38, 63%) showed no difference in their WRS compared to the unaided group. The HA group showed significantly better audiological performance than the unaided and SHAA groups in all HINT situations. The HA group showed the highest scores in the IOI-HA, but the SHAA users also showed significantly greater improvement than the unaided group. A comparison of individuals in the SHAA group between unaided and aided conditions found that 37% (14/38), 42% (16/38), 24% (9/38), and 32% (12/38) of users showed improvements in the ease of communication, reverberation, background, and aversiveness of sound subscales of the APHAB, respectively.

### Comparisons With Previous Research

de Sousa et al [34] investigated the electroacoustic and self-reported performance of SHAAs in the Google Play and Apple App stores. The Petralex app in the Android Samsung S6 showed a 3.9-dB improvement in the SNR at full noise reduction. The SNR became worse with no noise suppression. Of 5 participants, 4 agreed that conversations were easy to follow while using the app. However, all 5 participants preferred the HA to the SHAA. The study suggested that Petralex showed the best performance in noise reduction. de Sousa et al [34] also evaluated the subjective listening experience of participants using the Petralex app. However, they used only the electroacoustic analysis to evaluate noise reduction and investigated the subjective listening experience of only 5 participants.

Amlani et al [23] reported that SHAAs improved the perceived benefits and reduced the perceived barriers in first-time users of amplification and those who had quit using HAs. They conducted a survey before and after SHAA use. However, their study did not evaluate hearing performance with hearing tests, and there was no control group.

Reed et al [32] compared 5 personal sound amplification products (PSAPs) to conventional HAs for understanding speech in noise. Three of the PSAPs improved speech understanding for individuals with hearing loss, similar to the results obtained with HAs, whereas 1 product demonstrated little improvement and 1 product degraded speech understanding. This study suggested that the alternative devices to HAs, which are more affordable and easier to access, can improve speech understanding. Several studies have compared PSAPs to conventional HAs with varying results. However, only a few studies of SHAAs have been conducted.

Lin et al [35] reported that smartphone-bundled earphones can be used as PSAPs for mild-to-moderate hearing loss. They used AirPods Pro (Apple Inc) and compared it to 5 other PSAPs. In quiet conditions, there was no difference in speech perception between the AirPods Pro and the HAs, but they differed in noisy conditions. That study did not evaluate the performance of SHAAs and did not include a subjective satisfaction evaluation. Most studies, including this study, have suggested that HAs lead to the best performance in speech perception in noisy conditions.

Our previous study compared the electroacoustic characteristics of SHAAs to those of HAs and found that only a few performed reliably [19]. In another study, we evaluated the behavioral performance of a selection of currently available SHAAs in patients with mild hearing loss. However, it was an exploratory pilot study with only 7 participants [24].

Martinez-Beneyto et al [36] studied whether audiological smartphone apps can improve audiological performance in groups without hearing loss or groups with various grades of hearing loss. Most participants showed improvement in PTA and word recognition. They used the Apple iPhone 6S iOS (version 10.1.1) and the Sony MDR-EX15LP in-ear headphones. In our study, we used Android devices, and the audiological performance of the SHAA showed a significant difference according to the smartphone device and bundled earphone. Furthermore, that study was conducted among patients with mild-to-severe hearing loss. Although this study included only patients with mild-to-moderate hearing loss, we think that the findings could be generalized to those with more severe hearing loss. A comprehensive comparison with currently used methods is summarized in Table 3.

**Table 3.** Comparison with currently used methods.

Study	Purpose	Type of amplification	Test measure	Key findings
de Sousa et al [34]	Investigate electroacoustic and self-reported performance through various apps and smartphone manufacturers	4 SHAAs^a^ (Petralex, Super Ear, Earshot, and Hearing Aid Master) on Android devices 4 SHAAs (Petralex, Fennex, Mobile Ears, and Super Hearing Aid) on iPhone devices	Objective sound quality (latency and SNR^b^) and subjective listening experience	Petralex and Fennex on iPhone 6 showed the shortest latency and highest SNR improvement All SHAAs showed longer latency and lower SNR improvement on Android devices Participants preferred using HAs^c^ over SHAAs
Amlani et al [23]	Determine the feasibility of improving attitude during 4 weeks with an SHAA	1 SHAA (Ear Machine) with iPod Touch	N/A^d^	The SHAA can modify the perception toward amplification and reduce the perceived barriers in first-time users of amplification
Reed et al [32]	Compare PSAPs^e^ with a conventional HA for mild-to-moderate hearing loss	5 PSAPs (Sound World Solutions CS50+, Soundhawk, Etymotic BEAN, Tweak Focus, and MSA 30X Sound Amplifier) and 1 HA (Oticon Nera2)	AZBio sentence-in-noise task	3 PSAPs improved speech understanding, similar to the results with the HA 1 PSAP showed worse speech understanding than the unaided condition
Lin et al [35]	Examine electroacoustic properties of AirPods and compare hearing performance for mild-to-moderate hearing loss	5 PSAPs, 2 HAs (Oticon Opn 1 and Benafon MD 1), AirPods Pro, and AirPods 2	Mandarin HINT^f^	AirPod Pro met 4 PSAP standards No difference in speech perception between the AirPod Pro and HA in quiet conditions
Koo et al [24]	Evaluate the behavioral performance of 3 SHAAs	3 SHAAs (Ear Machine, Sound Amplifier, and Petralex)	REM^g^, warble-tone audiometry, WRS^h^, and the HINT (quiet and noise-front conditions)	HAs showed greater gain than SHAAs at 2 and 3 kHz in sound-field audiometry 6% showed improvement with Petralex in WRS No improvement was found with SHAAs in the HINT Some SHAAs were beneficial for patients with mild-to-moderate hearing loss
Martinez-Beneyto et al [36]	Assess whether SHAAs can improve audiological performance in patients without hearing loss or those with varying grades of hearing loss	1 SHAA (Petralex) with iPhone 6S	PTA^i^, WRS in quiet and noisy conditions, and a questionnaire	SHAA can improve word recognition and PTA 61% answered “good” or “excellent” for the app sound quality

a SHAA: smartphone-based hearing aid app.

b SNR: signal-to-noise ratio.

c HA: hearing aid.

d N/A: not applicable.

e PSAP: personal sound amplification product.

f HINT: hearing-in-noise test.

g REM: real-ear measurement.

h WRS: word recognition score.

i PTA: pure-tone audiometry.

### Strengths and Limitations

To the best of our knowledge, this is the first prospective, multicenter randomized controlled trial comparing an SHAA and a conventional HA. Hearing performance was evaluated for comprehensive objective functional gain and for subjective satisfaction.

In this study, we enrolled Android users who used their own smartphones. Although the performance varies depending on which app is used on different smartphone models, de Sousa et al [34] reported that SHAAs on iPhone 6 and iOS with wired earphones showed better SNR improvement and sound quality than SHAAs on Samsung S7. However, newly introduced devices and later versions of Android may show better amplification performance. Furthermore, Nguyen et al [19] found that most apps in iOS provided better electroacoustic performance than the corresponding Android versions. Another limitation of our study is a high dropout rate because of the COVID-19 pandemic, which hindered many patients from visiting. Finally, audiological performance was evaluated only after a 2-month trial, and 2 months may not be enough time to fully evaluate and clarify the long-term performance of HAs and SHAAs. Longer-term research is needed in the future.

### Conclusion

In our study, the SHAA demonstrated a significant benefit when compared to an unaided situation. Our results indicate that the SHAA could be a useful assistive device for patients with mild-to-moderate hearing loss in quiet conditions. In addition, people with poor access to hearing amplification devices can more easily download an SHAA at a more reasonable price than a conventional HA. Although, the SHAA performed poorly when compared with the HA at a conversational sound level and in noisy conditions, the future signal technology of SHAAs should improve and perform better in noisy environments.

## Supplementary material

10.2196/46911 Checklist 1 CONSORT-eHEALTH checklist (V 1.6.1).

## Abbreviations

APHAB: Abbreviated Profile of Hearing Aid Benefit
HA: hearing aid
HINT: hearing-in-noise test
IOI-HA: International Outcome Inventory for Hearing Aids
PSAP: personal sound amplification product
PTA: pure-tone audiometry
REM: real-ear measurement
SFA: sound-field audiometry
SHAA: smartphone-based hearing aid app
SNR: signal-to-noise ratio
SPL: sound pressure level
WHO: World Health Organization
WRS: word recognition score
